# The burden of Japanese encephalitis, the catch-up vaccination campaign, and health service providers’ perceptions in Myanmar: 2012–2017

**DOI:** 10.1186/s41182-020-00200-3

**Published:** 2020-03-05

**Authors:** Aung Ye Naung Win, Khin Thet Wai, Anthony D. Harries, Nang Thu Thu Kyaw, Tin Oo, Wint Phyo Than, Htar Htar Lin, Zaw Lin

**Affiliations:** 1 Epidemiology Research Division, Department of Medical Research, Ministry of Health and Sports, No. 5, Ziwaka Road, Dagon Township, Yangon, 11191 Myanmar; 2 International Union against Tuberculosis and Lung Disease, Paris, France; 3 London School of Hygiene and Tropical Medicine, London, UK; 4 Vector Borne Disease Control Program, Ministry of Health and Sports, Naypyitaw, Myanmar; 5 Expanded Program on Immunization, Ministry of Health and Sports, Naypyitaw, Myanmar; 6 WHO South East Asia Regional Office, New Delhi, India

**Keywords:** Myanmar, Japanese encephalitis, Children, Death, Catch-up vaccination campaign, Health service provider perceptions, SORT IT

## Abstract

**Background:**

Myanmar is endemic for Japanese encephalitis (JE) and has experienced several outbreaks in recent years. The vector-borne disease control (VBDC) program has collected hospital-based surveillance data since 1974. There is an urgent need to collate, analyze, and interpret the most recent information. The study aimed to describe (i) hospital-based JE cases and deaths between 2012 and 2017, (ii) a catch-up vaccination campaign in children in 2017, and (iii) health service provider perceptions about JE in one township in 2018.

**Methods:**

This was a cross-sectional study of cases, deaths, and catch-up childhood vaccinations using secondary data from program records and a survey database of health service provider perceptions.

**Results:**

Between 2012 and 2017, there were 872 JE cases and 79 deaths with a case fatality rate of 91 per 1000; 2016 was the year with most cases and deaths. Most cases (*n* = 324) and deaths (*n* = 37) occurred in children aged 5–9 years. Large case numbers were reported in delta and lowland regions (*n* = 550) and during the wet season (*n* = 580). The highest case fatality rates were observed in the hills and coastal regions (120 and 112 per 1000, respectively). Nationwide coverage of the catch-up JE vaccination campaign among 13.7 million eligible children was 92%, with coverage lower in the hills and coastal regions (84%) compared with delta and lowland regions and plains (94%). More vaccinations (65%) occurred through school-based campaigns with the remainder (35%) vaccinated through community-based campaigns. Structured interviews in one township showed that service providers (*n* = 47) had good perceptions about various aspects of JE, although perceived benefits of specific vector control measures were poor: spraying/fumigation (38%), garbage removal (36%), larvicide use (36%), and drainage of standing/stagnant water (32%).

**Conclusion:**

The catch-up vaccination campaign was a successful response to high JE case numbers and deaths in children. However, ongoing surveillance for JE needs to continue and be strengthened to ensure comprehensive reporting of all cases, more knowledge is needed on disability in JE survivors, and all attempts must be made to ensure high percentage coverage of vaccination through routine and catch-up campaigns.

## Background

Japanese encephalitis (JE) is an acute inflammatory disease caused by the Japanese encephalitis virus (JEV), 1 of 70 viruses in the *Flavivirus* genus of the family Flaviviridae. It is the main cause of viral encephalitis in Asia and a large area of the Western Pacific with an estimated 68,000 clinical cases of encephalitis every year [1]. It is mosquito-borne, with the main vector in tropical and subtropical regions being *Culex tritaeniorhynchus*, which bites mainly at night [1]. The virus is maintained and amplified by intermediate hosts, primarily pigs and wading birds. Transmission to humans not only occurs most frequently in agricultural areas such as farms and rice paddies, but also occurs in peri-urban or urban areas with appropriate ecological conditions. More than 3 billion people are at risk of transmission [1]. The occurrence of JE is seasonal in temperate climes with the main period of risk being May to October, but in tropical regions, the risk is all year round.

In endemic countries, like Myanmar, symptomatic JE is generally reported in children. Of individuals who are infected, less than 1% will progress to encephalitis [2]. Of the 68,000 estimated clinical cases of encephalitis each year, between 20–30% die and 30–50% of survivors have significant neurological or psychiatric sequelae [3]. Individuals who live in or have traveled to a JE-endemic area and develop encephalitis are considered a suspected JE case, and confirmatory diagnosis is through laboratory testing of serum or cerebrospinal fluid (CSF). There is no specific antiviral treatment for JE and treatment is supportive. Safe and effective vaccines are available to prevent disease, with new generation JE vaccines gradually replacing those derived from mouse brains [1, 2, 4, 5]. It seems clear that where vaccination coverage is poor, there is a higher risk of JE especially among children [6], but even in areas where JE vaccination programs have developed or are becoming established, the incidence of JE can remain quite substantial [7].

JE was first reported as an outbreak in Tarchileik Township, Shan State, Myanmar, in 1974, and over the next 5 years, Shan State reported outbreaks on an annual basis. More recently and from 2010, JE outbreaks have been reported from 13 states/regions of Myanmar, with just over 50% of cases occurring in rural populations [8–11]. In 2016, there were serious outbreaks of JE, and in mid-August 2017, the Department of Public Health, Myanmar, reported 404 cases of JE from 14 states/regions and Nay Pyi Taw Council Territory. Despite the growing importance of JE in Myanmar, the attention of service providers to provide an adequate coordinated response in a “one health approach to reduce zoonotic epidemic threats” has been limited. There have been gaps in public–private partnerships and poor coordination between health authorities, veterinarians, and the public in terms of education campaigns, vector control measures, vaccination of at-risk populations, and epidemic preparedness and response.

To address these gaps, in 2017, a nationwide JE vaccination catch-up campaign was started for schools and the community. Data were also collected from selected service providers about their knowledge, attitudes, and perceptions towards the disease and its prevention. The published and reported data on JE in Myanmar is local and fragmented [8, 9, 11], and there is a great need to have a comprehensive situational analysis of country-wide hospital-based cases over a 6-year period. This data, combined with information about the recent 2017 catch-up vaccination campaign and perspectives from service providers, will be valuable for the vector-borne disease control (VBDC) program and the Expanded Programme on Immunization (EPI) under the Department of Public Health, Ministry of Health and Sports, so that they can develop a new coordinated strategic plan to successfully reduce JE transmission in the country.

The aim of this study, therefore, was to describe the burden of hospital-based Japanese encephalitis, the vaccination campaign, and health service provider perceptions about the disease and its prevention in Myanmar. Specific objectives were to describe (i) the hospital-based cases with JE in Myanmar, associated characteristics, and deaths over a 6-year period from 2012 to 2017; (ii) the number of children vaccinated in the JE vaccination catch-up campaign in 2017; and (iii) health service provider perceptions about JE, its transmission, and prevention in one township, Bago Region in 2018.

## Methods

### Study design

This was a cross-sectional descriptive study using secondary data from program records and one survey database.

### Setting

#### General setting

Myanmar is located in the South East Asia Region, neighboring the Republic of China on the north and northeast, Laos on the east, Thailand on the southeast, Bangladesh on the west, and India on the northwest. The country is separated administratively into Nay Pyi Taw Council Territory and 14 states and regions. It consists of 74 districts, 330 townships, 398 towns, 3065 wards, 13,619 village tracts, and 64,134 villages. The main terrestrial features of the country are the delta region and the central plain surrounded by mountains. Myanmar enjoys a tropical climate with three different seasons: rainy, cold, and hot. In 2014, Myanmar had a population of about 52 million people with an urban–rural population ratio of 30:70 [12]. It has an area of 676,577.2 km^2^ and a population density per square kilometer of 76.1 [12].

#### JE and its prevention and control in Myanmar

In Myanmar, JE was included as one of the five principal epidemic diseases in 1976. However, in 1989, JE lost this status although in 1995 it was made a notifiable disease. From 2000 onwards, JE was included in the meningitis/encephalitis disease entity in the National Health Management Information System (HMIS), and surveillance of the acute encephalitis syndrome (AES) was initiated in 2007. The prevention and control of JE outbreaks involve a mixture of activities that include case studies, active case finding, serological surveys, entomological studies, adult vector control measures, and health education in schools and the community [10]. The current National JE Control Strategy from the Department of Public Health for prevention and control of JE is in line with the strategy agreed at the sixth bi-regional meeting on JE for the WHO South-East Asia and Western Pacific regions held in May 2014 [13], the key components of which are (i) adult mosquito control, (ii) mosquito breeding control, and (iii) human vaccination. The two components missing in the Myanmar health sector from the WHO strategy are vaccination of pigs and improved living conditions.

#### JE case surveillance

Serological surveys have been conducted in Myanmar since 1968 when the existence of the disease in the country was first discovered [10]. The earliest clinical case was recorded in 1974 in Tarchileik Township in the Eastern Shan State. Annual outbreaks of JE with considerable morbidity and mortality occurred between 1974 and 1979 with case fatality rates ranging between 50 and 100%. Between 1974 and 2011, thirty-eight townships of eight states and seven regions reported a total of 259 clinical cases of JE with 106 deaths, giving an overall case fatality of 41%. In 2012, seven townships of one state (Rakhine) and two regions (Yangon and Tanintharyi) reported 13 cases of Japanese encephalitis with no deaths, and in all these areas, apart from Hpa-an, Kayin State, the disease was serologically confirmed. In Kyaing Tone and Taunggyi of Shan State, JE outbreaks occurred over four consecutive years from 2012 to 2015. JE case surveillance is currently conducted jointly by the Department of Public Health and the Department of Medical Services in line with national guidelines [14].

#### JE case management, vector control measures, and recording/reporting

Patients admitted to hospital with AES are suspected to have JE. In these situations, hospital staff work in collaboration with teams from the VBDC program to collect blood (3 ml of venous blood) and cerebrospinal fluid (0.5 ml of CSF) specimens, and these are sent to the National Health Laboratory in Yangon, which was under the Department of Public Health up to 2015, since when it has been under the Department of Medical Services. Diagnostic confirmation is done through serological testing and CSF examination for JE viral-specific antibodies using the JE IgM ELISA Test Kit “JE Detect™ IgM Antibody Capture ELISA (Mac-ELISA)” from InBios International, Inc., WA, USA [15]. Because JE virus can cross-react with dengue virus on antibody testing, the National Health Laboratory follows WHO Guidelines for confirming JE virus infection [16]. This works as follows. Any serum showing a positive JE IgM test result is retested using InBios Dengue Detect IgM Antibody Capture ELISA [17]: if this test is negative, the serum is reported as JE positive, and if the test is positive, the serum is reported as dengue positive. Serum with equivocal results from the dengue testing is retested with the reporting algorithm similar to the above except that a second equivocal test is reported as JE positive. All JE-positive and JE equivocal tests are sent to the WHO regional reference laboratory for confirmatory testing. Standard operational definitions for AES and JE are shown in Table 1 [18]. Confirmed JE cases are notified to the Department of Public Health that then authorizes JE source reduction measures. VBDC officers take responsibility for instituting adult mosquito control measures (space spraying/thermal fogging using malathion insecticide) in the urban ward or village where the index patient resides.

**Table 1:** Operational definitions for acute encephalitis syndrome and Japanese encephalitis

SN	Variables	Operational definition
1	Acute encephalitis syndrome (AES)	A patient with fever (> 38 °C) and changes in mental status, abnormal movements, seizure, tremor, or spastic paralysis.
2	Japanese encephalitis (JE)	A case of AES with IgM against the Japanese encephalitis virus in the serum and/or cerebrospinal fluid. If IgM is detected just in the serum, the diagnosis is probable JE. If IgM is detected in the serum and cerebrospinal fluid, the diagnosis is confirmed JE. The JE Detect™ IgM Antibody Capture ELISA (MAC-ELISA) from InBios International, Inc., Seattle, WA, USA, was used for laboratory testing.

Records of JE cases are kept at the hospital, and a focal person from the VBDC team collects these on a weekly basis through fax, electronic mail, and phone calls. During JE outbreaks, the collection of reports is daily rather than weekly. These case records are collated by focal persons of the VBDC team who transfer the data upwards from township to district to state/region and finally to the Central VBDC Programme at the Department of Public Health. The Central VBDC Programme reports weekly to the Ministry of Health and Sports, and from there, reports are sent to WHO SEARO through the WHO Country Office of Myanmar.

#### JE vaccination campaign

In 2017, the EPI undertook a nationwide JE vaccination catch-up campaign supported by funds from the Global Alliance for Vaccines and Immunizations (GAVI). The purpose of this was to provide high immunity to JE before JE vaccines were incorporated onto the national routine immunization schedule in 2018. Technical support was provided by WHO, the United Nations Children’s Fund (UNICEF), and the Programme for Appropriate Technology in Health (PATH). Nearly 14 million children (aged 9 months to 15 years) were targeted using the WHO pre-qualified live attenuated (SA 14-14-2) JE vaccine, which is the same one to be used in the routine immunization schedule. The campaign was launched in two phases: a school-based phase targeting 5–15-year-old students between 15 and 23 November 2017 and a community-based phase targeting children aged 9 months to 5 years (out-of-school) between 11 and 20 December 2017 [19].

#### Service provider perceptions in Letpadan Township, Bago Region

Bago Region includes 28 townships and about 4.8 million inhabitants. There are three distinct seasons in the region that are hot (March to May), wet (May to October), and cool (November to February). Between 2012 and 2017, there were 121 reported cases of JE and eight associated deaths. Letpadan Township was one of the five townships in Bago Region with a high number of cases and deaths [20]. In January 2018, a team from the Department of Medical Research conducted structured interviews in Letpadan Township following the JE vaccination catch-up campaigns. These interviews were carried out among service providers which included VBDC staff, health assistants, lady health visitors, public health supervisors, and midwives. The selection of participants for interviews was purposive so as to have comprehensive representation at the township level. Ethics approval for this particular survey was obtained from the Ethics Review Committee of the Department of Medical Research (Ethics/DMR/2018/008).

### Study population

The study included (i) all patients diagnosed with JE in hospitals in Myanmar between 2012 and 2017, (ii) children vaccinated during the catch-up campaign in 2017 in both the school-based phase and community-based phase, and (iii) health service providers who were interviewed in the public sector in Letpadan Township, Bago Region.

### Variables, data sources, and data collection

Data variables for the study included the following: for hospital-recorded cases of JE and deaths—year, month, age group, gender, urban/rural residence, season, and ecological area of the country for each case. The definition of ecological areas was adapted from Oo et al. [21] and is shown in Table 1. The source of data was the VBDC electronic EXCEL database for national aggregate data; for vaccinated children—ecological region, children eligible for vaccination, children vaccinated, children vaccinated during the school-based campaigns, and children vaccinated during the community-based campaigns. The source of data was the electronic EXCEL database for aggregate data obtained from the 2017 EPI records and donor reports; for health service provider perceptions—these were structured wide-ranging questions related to perceptions, attitudes, and opinions about JE and its prevention. The data source was secondary data from the survey database of the DMR that focused on Letpadan Township, Bago Region.

### Analysis and statistics

Data were extracted and cleaned in an EXCEL file and exported to EpiData software (version 2.2.2.182 for analysis, EpiData Association, Odense, Denmark). A descriptive analysis was performed on JE cases, JE deaths, and results of the vaccination campaign using frequencies and proportions. Hospital-based JE case fatality rates were calculated by the number of deaths per 1000 JE cases per year. Cases and deaths were analyzed in relation to certain characteristics such as age group, gender, urban/rural residence, season, and ecological region. Maps of JE cases and deaths at state and regional levels were generated through a geographical information system using QGIS [22]. Perceptions, attitudes, and opinions of health service providers were analyzed by frequencies and percentages.

## Results

### Hospital-recorded cases of JE and JE deaths

**Fig. 1:**
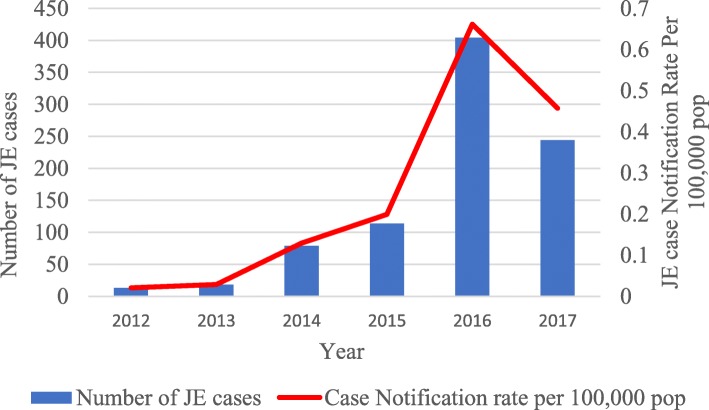
Annual hospital-reported JE cases and case notification rates per 100,000 population in Myanmar: 2012 to 2017

**Fig. 2:**
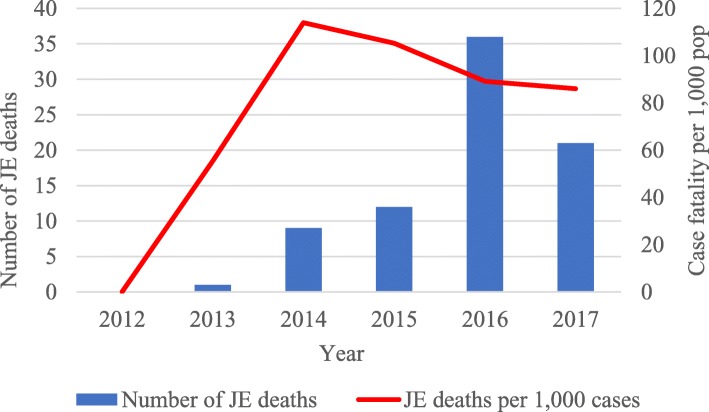
Annual hospital-reported JE deaths and case fatality rates in Myanmar: 2012 to 2017

The annual number of hospital-reported cases of JE and associated deaths are shown in Figs. 1 and 2. During the 6-year period, there was a total of 872 JE cases and 79 JE deaths, with an overall case fatality rate of 91 per 1000 cases. In 2016 and 2017, there was an increase in the number of cases at 404 and 244, respectively, while during the other 4 years, the number of cases ranged from 13 to 114. Case notification rates per 100,000 were similar to the pattern seen with the absolute number of cases. The distribution of JE deaths each year mirrored the distribution of cases. The case fatality rate was highest in 2014 at 114 per 1000 JE cases, while in the other years, this ranged between 0 and 100 per 1000 cases.

Demographic characteristics, seasonal changes, and the ecological distribution of all hospital-recorded JE cases and deaths between 2012 and 2017 are shown in Table 2. Approximately, 85% of all cases were in children, with those aged 5–9 years being the predominant age group affected. The highest case fatality rates were also observed in the age group 5–9 years. Males were more likely to have JE compared to females, but case fatality was higher in females. Those residing in rural settings were at higher risk of JE and death compared with urban settings. The majority of cases and deaths, and the highest case fatality rates, occurred in the wet season from May to September. Most cases (63%) and deaths (53%) occurred in persons living in the delta and lowland areas although case fatality was highest in those residing in the hills.

**Table 2:** Operational definitions ecological regions in Myanmar

SN	Variables	Operational definition
1.1	Delta and lowland (heavy rainfall more than 2500 mm)	Ayeyawady, Yangon, and Bago regions; Mon and Kayin states
1.2	Hills (moderate to heavy rainfall)	Kachin, Kayah, Chin, and Shan states
1.3	Coastal (heavy rainfall more than 2500 mm)	Rakhine State and Tanintharyi Region
1.4	Plains (uneven topography and rainfall less than 1000 mm)	Magway, Mandalay, Sagaing, and Nay Pyi Taw regions

Adapted from [19]

**Fig. 3:**
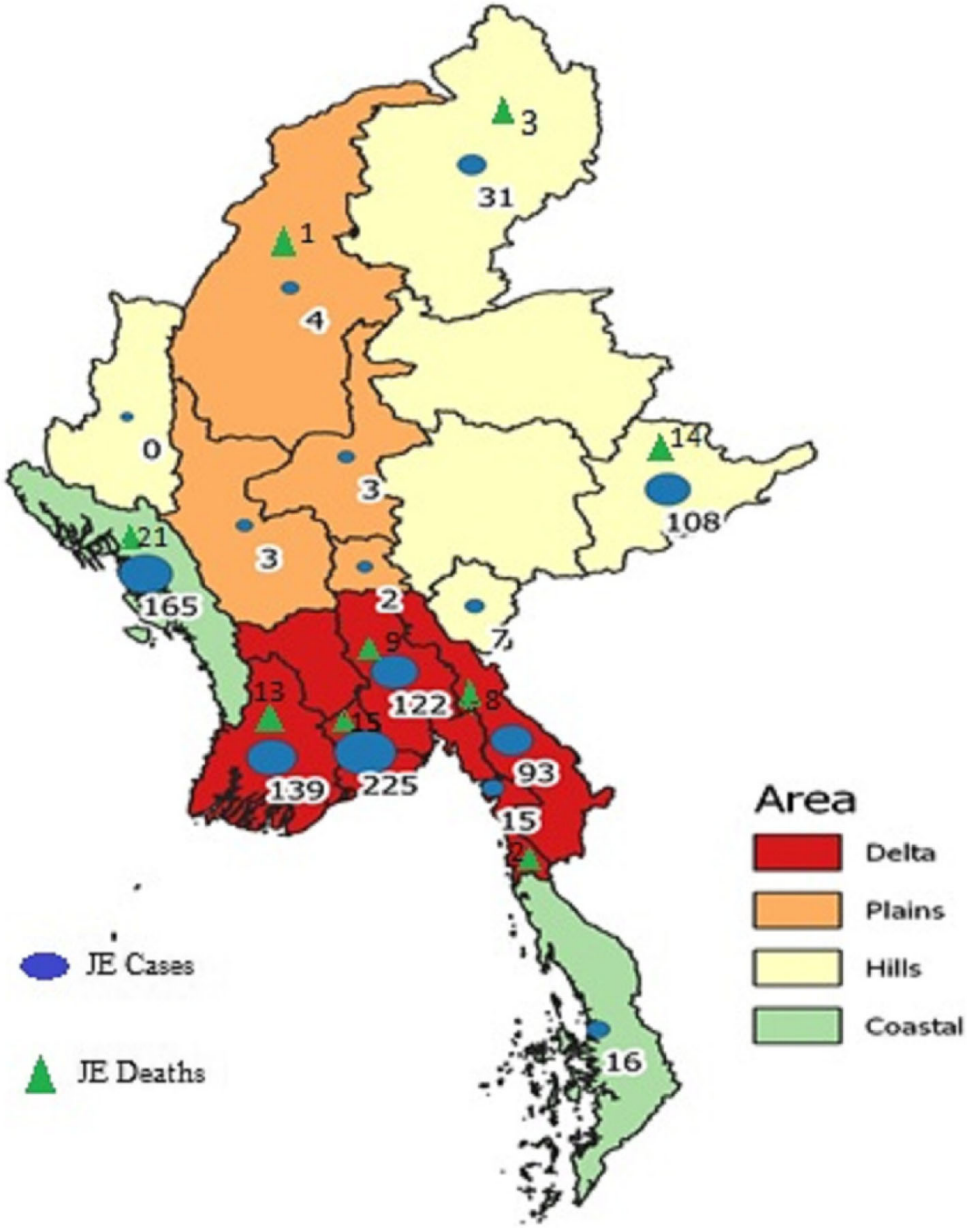
Distribution of all hospital-reported JE cases and deaths in Myanmar between 2012 and 2017

Figure 3 is a map that shows JE cases and deaths in relation to the four ecological regions of the country. The five main hot spots for JE cases were Yangon, Ayeyawady, and Bago regions followed by Rakhine and Shan states. The main hot spots for JE deaths were Rakhine and Shan states followed by Yangon, Ayeyawady, and Bago regions.

### Childhood vaccination campaign for 2017

Numbers and proportions of children vaccinated against JE during school-based and community-based catch-up campaigns in 2017 for the whole country and by ecological region are shown in Table 3. Nationwide coverage of JE vaccination among eligible children was 92%. Vaccination coverage was lower in the hills and coastal regions (84%) compared to delta and lowland regions and the plains (94%). Nearly two thirds of the children were vaccinated through school-based campaigns with the remainder vaccinated through community-based campaigns. These ratios of school-based to community-based campaigns were similar in the delta and lowlands, plains, and coastal regions, while in the hilly regions, school-based campaigns were fewer and constituted 58% of vaccination coverage.

**Table 3:** Demographic characteristics, seasonal changes, and the ecological distribution of hospital-recorded JE cases, JE deaths, and case fatality rates in Myanmar between 2012 and 2017

Characteristics	JE cases, *n* (%)	JE deaths, *n* (%)	Case fatality per 1000 JE cases
Total	872 (100)	79 (100)	91
Age group in years			
< 1	21 (2)	1 (1)	48
1–4	198 (23)	14 (18)	71
5–9	324 (37)	37 (47)	114
10–14	199 (23)	16 (20)	80
≥ 15	130 (15)	11 (14)	85
Gender			
Male	484 (56)	34 (43)	70
Female	388 (44)	45 (57)	116
Residence			
Urban	293 (34)	21 (27)	72
Rural	579 (66)	58 (73)	100
Season			
Hot (February to April)	169 (19)	7 (9)	41
Wet (May to September)	580 (67)	63 (80)	109
Cool (October to January)	123 (14)	9 (11)	73
Ecological region			
Delta and lowland	550 (63)	42 (53)	76
Hills	142 (16)	17 (22)	120
Coastal	170 (20)	19 (24)	112
Plains	10 (1)	1 (1)	100

Percentages are column percentages

*JE* Japanese encephalitis

### Perceptions, attitudes, and opinions of health service providers

Perceptions, attitudes, and opinions of 47 health service providers about JE infection in Letpadan Township, Bago Region, in January 2018, are shown in Table 4. Although 94% of health service providers accepted JE as a serious illness, their perceived risk about contracting JE and their increased risk of being a resident in the particular locality were just over 50% (Table 5). Over 90% of the health service providers showed a positive attitude towards the prevention of JE through compliance with VBDC guidelines. However, less than 40% believed that specific measures to prevent JE would be useful such as spraying to reduce the adult mosquito population, removing garbage dumps, using larvicides, and improving drainage. Over 90% of health service providers believed that epidemic surveillance of JE in humans and pigs and removal of the JE vectors (mosquitoes) would reduce JE transmission. Three quarters believed that JE vaccination should be given to children aged 9 months to 15 years. While less than 40% stated the need for stakeholders to be updated on JE, there was strong support for villagers to be educated about the disease through either community or school-based education sessions.

**Table 4:** Numbers and proportions of children vaccinated against JE during school-based and community-based catch-up campaigns in 2017 in Myanmar for the whole country and by ecological region

Ecological region of Myanmar	Children eligible for JE vaccination^a^, *n*	All children vaccinated, *n* (%)^b^	Children vaccinated during school-based campaigns, *n* (%)^c^	Children vaccinated during community-based campaigns, *n* (%)^c^
Whole country	13,783,983	12,584,351 (92)	8,157,502 (65)	4,426,849 (35)
Delta and lowland	5,749,759	5,381,590 (94)	3,502,303 (65)	1,879,287 (35)
Plains	4,259,661	4,027,788 (95)	2,736,234 (68)	1,291,554 (32)
Hills	2,466,304	2,075,633 (84)	1,203,625 (58)	872,008 (42)
Coastal	1,308,260	1,099,340 (84)	715,340 (65)	384,000 (35)

Source: Expanded Program on Immunization, Myanmar

*JE* Japanese encephalitis

^a^Children between 9 months to 15 years of age eligible for JE vaccination

^b^Percentage of children eligible for JE vaccination

^c^Percentage of all children vaccinated

**Table 5:** Health service providers’ perceptions, attitudes, and opinions about JE infection and its prevention in Letpadan Township, Bago Region, Myanmar, in January 2018

Perceptions, attitudes, and opinions of health service providers	*n*	(%)
Health workers who were interviewed	47	
Health workers who agreed with the following statements:		
Perceptions:		
JE is a serious illness.	44	(94)
Pig farmers are at risk of JE.	41	(87)
Health workers are at risk of JE.	27	(57)
Living in this area increases the risk of getting JE.	26	(55)
Attitudes:		
JE infection can be prevented by using VBDC guidelines.	44	(94)
JE can be prevented by spraying or fumigation.	18	(38)
JE can be prevented by removal of garbage dumps.	17	(36)
JE can be prevented by using larvicides.	17	(36)
JE can be prevented by draining clean water.	15	(32)
Opinions:		
Health workers should undertake epidemic surveillance in both humans and pigs.	43	(92)
Removal of the JE vector is a good strategy to reduce JE transmission.	43	(92)
Stakeholders should be well informed with updates of JE.	29	(38)
JE vaccination should be given to children age 9 months to 15 years.	35	(75)
The most preferred way to educate villagers more about JE is through:		
a) Community-based education sessions	21	(45)
b) School-based education sessions	20	(43)
c) Facility-based education sessions	6	(13)

*JE* Japanese encephalitis, *VBDC* vector-borne diseases control

## Discussion

This is the first study from Myanmar assessing the national burden and characteristics of hospital-reported JE cases and deaths over a 6-year period, documenting the results of the 2017 catch-up vaccination campaign in children and describing health service providers’ perceptions of JE and its prevention. There were a number of important findings.

There were nearly 900 reported cases of JE with a case fatality rate of 91 per 1000. The majority of cases and deaths occurred in children, particularly those aged 5–9 years. The year 2016 was the worst for absolute numbers of cases and deaths, which led to the catch-up vaccination campaign in 2017. This was an ecological study, and we therefore did not capture any information about morbidity after recovery from the infection. Previous reviews point to significant neurological sequelae being present in up to 50% of survivors [3, 23], so while this disease is less common in the country than dengue [21], it may nevertheless be very demanding on health service care and resources. While care is currently supportive, there is an ongoing work exploring the prospects for direct-acting antiviral treatment, especially with the use of nucleoside and nucleotide reverse transcriptase inhibitors [24, 25]. Two studies in India have evaluated the use of minocycline, and while there was some evidence of reduced symptom duration and hospital stay, there were no survival benefits [26, 27]. Finally, there has been some preliminary work on the use of intravenous immunoglobulin in Nepal [28], again without proof yet of survival benefit.

The finding that JE cases were more frequent in the wet season and in delta and lowland regions (with paddy fields and areas prone to flooding) aligns with current knowledge about transmission of JE in Asian countries [29, 30]. There are seasonal correlations in rural areas between mosquito abundance, JE virus seroconversion in pigs, and concurrent human JE outbreaks [29]. In the wet season, the widespread and timely implementation of mosquito larval control measures and space spraying of townships and wards with insecticides may help to contain mosquito proliferation and reduce human outbreaks. However, a recent report of increasing resistance of two important JE vectors (*Culex tritaeniorhymchus* and *Culex vishnui*) to DDT, malathion, and deltamethrin in India is of concern to the region [31]. Case fatality was higher in the hills, similar to what was found 2 years ago with dengue [21]. This may be because health facilities are not as well-resourced in these areas, health care workers are less experienced in providing supportive care for encephalitis, and there is poor immunity of the population to the JE virus.

It was encouraging to see the high uptake of JE vaccination in the 2017 catch-up campaign, especially in the delta and lowland areas where the burden of JE is highest. The SA 14-14-2 live attenuated JE vaccine, whether used on routine or campaign basis, has shown excellent and durable efficacy in China, Nepal, and South Korea [32], although results have been less impressive in India maybe as a result of cross-contamination from non-vaccinated to vaccinated districts [33]. Good surveillance will be needed in Myanmar to determine whether morbidity and mortality from JE are reduced through this strategy of a one-off catch-up vaccination campaign followed by routine immunization or whether further catch-up campaigns may be necessary, as is the case in Nepal [34].

Finally, the structured interviews in the township showed that service providers had a reasonable degree of knowledge about the serious nature of JE, the need for epidemic surveillance in humans and pigs, the importance of JE vaccination of children and about the value of regularly updated education of rural populations about JE. Knowledge related to specific vector control measures was poor, and this needs to be improved. There have been no recent published studies on knowledge, attitude, or practice of service providers in relation to JE in Myanmar with which to compare these findings.

The strengths of the study were the hospital-recorded cases and deaths of JE from all health facilities in all states and regions of the country over a 6-year period, making the findings representative of what is happening in the country. The conduct and reporting of this observational study were also in line with internationally recommended Strengthening the Reporting of Observational Studies in Epidemiology (STROBE guidelines) [35].

However, there were a number of limitations. First, we do not know how well or comprehensively all children in the country with acute encephalitis syndrome (AES) were investigated for JEV. In Mandalay hospital, four of 123 children presenting with acute encephalitis had JEV diagnosed in their CSF by neutralization tests and/or virus isolation [11], but if the tests are not performed on all children presenting with AES, the diagnosis will be missed. While it is likely that children with AES are admitted to the hospital given the serious nature of this condition, cases may develop quickly and die in the community. Our hospital-based recording system may therefore have underestimated the true burden of JE in the country. Second, we also had no information about the clinical profile of JE patients, the time that they spent in hospitals and among the survivors the degree of disability and dysfunction, so the burden of disease on the health care services is not known. Third, while the vaccination uptake among eligible children was high, we do not know the reasons why some children, particularly in the hills and coastal areas, did not receive the vaccination. This is important because widespread and complete vaccination coverage is needed to control this disease [23].

Despite these limitations, there are a number of programmatic implications. First, ongoing surveillance of hospital-based cases and deaths must continue and be strengthened with oversight to ensure that all AES cases are fully investigated for JE. Second, Myanmar should undertake a prospective evaluation of the neurological sequelae and disability resulting from JE and use this locally generated evidence to better inform the health care services and the general public about the seriousness of the disease and the need for its prevention. In this regard, the country could consider whether to use a new outcome score based on a 15-item questionnaire which has been successfully developed and tested by the WHO to assess the severity of disability resulting from JE in children and whether the child is likely to be dependent [36].

Third, every attempt must be made to ensure that the SA 14-14-2 live attenuated JE vaccine is fully incorporated into childhood routine immunization schedules, with ongoing surveillance needed to determine whether additional catch-up vaccination campaigns are needed, as is the case in Nepal [34]. Ultimately, JE is a vaccine-preventable disease, and high percentage coverage with routine as well as catch-up campaigns is the intervention most likely to control and even eliminate the disease. Unfortunately, there has been a gradual shift in JEV genotypes in Asia over the years, especially from genotype III to genotype I [37]. Almost all currently available vaccines are developed against the genotype III strains of JE, so this genotypic shift raises concerns about vaccine efficacy. Studies have shown that these vaccines are effective against genotype I [38], but less so against genotype V [39]. In light of these findings, Myanmar needs to decide how much effort and investment is also needed to vaccinate domestic pigs and undertake vector control. This would be in line with a coordinated “one health approach.” JEV in adult pigs does not result in symptomatic disease, but pigs can suffer from significant reproductive problems that include abortion, still-birth, and birth defects [40]. Protective vaccines are available for both pigs and horses [40].

## Conclusion

This study has described the burden of hospital-recorded JE in Myanmar between 2012 and 2017. The disease was concentrated in children and seen particularly in the delta and lowland regions and during the wet season. Overall case fatality was 91 per 1000, with high rates observed in children aged 5–9 years and people residing in hills and coastal regions. The catch-up JE vaccination campaign through school-based and community-based phases was successful with an overall 92% coverage although this was lower in the hills and coastal regions. Structured interviews in one township showed that service providers had a reasonable degree of knowledge about various aspects of JE, although this was poor with respect to specific vector control measures. For the future, ongoing surveillance for JE needs to continue and be strengthened, more knowledge is needed on disability in JE survivors, and all attempts must be made to ensure high percentage coverage of vaccination through routine and catch-up campaigns.

## Data Availability

All data generated or analyzed during this study are included in this published article.

## Abbreviations

CFR: Case fatality rate
CSF: Cerebrospinal fluid
EPI: Expanded Program on Immunization
JE: Japanese encephalitis
VBDC: Vector-borne disease control
WHO: World Health Organization
